# Major Depressive Disorder is Associated with Impaired Mitochondrial Function in Skin Fibroblasts

**DOI:** 10.3390/cells9040884

**Published:** 2020-04-04

**Authors:** Kerstin Kuffner, Julian Triebelhorn, Katrin Meindl, Christoph Benner, André Manook, Daniel Sudria-Lopez, Ramona Siebert, Caroline Nothdurfter, Thomas C. Baghai, Konstantin Drexler, Mark Berneburg, Rainer Rupprecht, Vladimir M. Milenkovic, Christian H. Wetzel

**Affiliations:** 1 Department of Psychiatry and Psychotherapy, University of Regensburg, 93053 Regensburg, Germany; kerstin.kuffner@ukr.de (K.K.); thomas.baghai@medbo.de (T.C.B.);; 2 Department of Dermatology, University Hospital Regensburg, 93053 Regensburg, Germany

**Keywords:** major depression, skin fibroblasts, mitochondria, bioenergetics, oxidative phosphorylation, adenosine triphosphate, calcium imaging, mitochondrial membrane potential, mitochondrial DNA copy number

## Abstract

Mitochondrial malfunction is supposed to be involved in the etiology and pathology of major depressive disorder (MDD). Here, we aimed to identify and characterize the molecular pathomechanisms related to mitochondrial dysfunction in adult human skin fibroblasts, which were derived from MDD patients or non-depressive control subjects. We found that MDD fibroblasts showed significantly impaired mitochondrial functioning: basal and maximal respiration, spare respiratory capacity, non-mitochondrial respiration and adenosine triphosphate (ATP)-related oxygen consumption was lower. Moreover, MDD fibroblasts harbor lower ATP levels and showed hyperpolarized mitochondrial membrane potential. To investigate cellular resilience, we challenged both groups of fibroblasts with hormonal (dexamethasone) or metabolic (galactose) stress for one week, and found that both stressors increased oxygen consumption but lowered ATP content in MDD as well as in non-depressive control fibroblasts. Interestingly, the bioenergetic differences between fibroblasts from MDD or non-depressed subjects, which were observed under non-treated conditions, could not be detected after stress. Our findings support the hypothesis that altered mitochondrial function causes a bioenergetic imbalance, which is associated with the molecular pathophysiology of MDD. The observed alterations in the oxidative phosphorylation system (OXPHOS) and other mitochondria-related properties represent a basis for further investigations of pathophysiological mechanisms and might open new ways to gain insight into antidepressant signaling pathways.

## 1. Introduction

Major depressive disorder (MDD) is a debilitating mental disorder with a lifetime prevalence of 15–20%, affecting about 320 million people worldwide (World Health Organization, 2019). The molecular mechanisms underlying the cause and the progress of this complex disease are still not completely understood. Hypotheses argue for a combination of neurobiological factors, which affect cellular function and resilience, thereby increasing the risk for MDD, and environmental (psychosocial) stress. This in turn is shown to be associated with the onset and severity of depressive episodes [1]. Involved molecular mechanisms include dysregulation of monoaminergic, glutamatergic and/or γ-aminobutyric acid (GABA)ergic neurotransmission as well as reduced neuroplasticity and neurogenesis as a consequence of impaired Brain-derived neurotrophic factor signaling (reviewed in [2]). Moreover, reduced bioenergetic function, which renders the cells vulnerable to stress, especially during increased metabolic demands, has been considered as an important risk factor for psychiatric disorders [3,4,5].

In the present study, we focus on bioenergetic aspects of MDD based on the hypothesis that mitochondrial dysfunction contributes to the etiology of the disease. Thus, we aim to identify and characterize the molecular pathomechanisms related to mitochondrial dysfunction and bioenergetic imbalance in a human cellular model of major depression. MDD is increasingly viewed as an illness of the mind as well as the body [6,7]. There are already reports of mitochondria-related effects in fibroblasts [7], muscle [8], peripheral mononuclear blood cells [9], and platelets [10,11] of depressed patients, suggesting that mitochondria-related pathomechanisms associated with MDD can be identified and studied in peripheral non-neuronal cells. To establish such a human model, we grew fibroblast cultures from skin biopsies in patients with major depressive disorder and from gender- and age-matched healthy control individuals. After several cell divisions in vitro, the confounding variability in these samples on the basis of the subjects’ hormones, life style or medication use, should be virtually eliminated [7]. As a measure for mitochondrial function/dysfunction, we analyzed the ATP-content, the function of the electron transport chain (ETC) by investigating the mitochondrial oxidative phosphorylation system (OXPHOS) and the mitochondrial membrane potential (MMP) as well as the cytosolic Ca^2+^ homeostasis in cultured human skin fibroblasts derived from depressed patients or non-depressed controls. Since stressful life events are associated with the onset and severity of major depression, we additionally set out to investigate whether aberrant adaptive responses to in vitro stressors are detectable at the cellular/mitochondrial level. Thus, we grew the fibroblast cultures under control conditions, but also treated the cells with glucose-free/galactose-containing medium or with dexamethasone to induce metabolic or hormonal stress, respectively.

## 2. Materials and Methods

### 2.1. Skin Biopsies and Primary Human Fibroblast Cultivation

Primary fibroblast lines were obtained by skin biopsy at the Department of Dermatology, University Hospital of Regensburg, Regensburg, Germany. Biopsy material of healthy skin (ø4 mm) was cut into smaller pieces, put into six wells of a 6-well plate (Sarstedt, Nümbrecht, Germany), attached for 5–7 min and covered with primary fibroblast medium (PrimFibM), consisting of 500 mL Dulbecco’s modified Eagle’s medium (DMEM)/F12 (Gibco, Life Sciences, Carlsbad, CA, USA) supplemented with 10% fetal calf serum (Sigma-Aldrich, St. Louis, MO, USA) and 1% Antibiotic-Antimycotic Solution (Sigma-Aldrich, St. Louis, MO, USA). The primary fibroblasts determined passage 0 were cultured for 2–3 weeks under standard conditions (37 °C and 5% CO_2_) until they reached confluency. The cells were split and transferred into 6-well plates for experimental purposes or into T75 flasks for further growth.

### 2.2. Passaging, Freezing and Thawing

At about 80–90% of confluency, cells were split by incubation in 1 mM **ethylenediaminetetraacetic acid** (EDTA) (Invitrogen by Life Technologies, Carlsbad, CA, USA) in phosphate-buffered saline (PBS, Gibco by Life Technologies; Carlsbad, CA, USA) for 15 min at 37 °C, and enzymatically detachment by Trypsin (Sigma-Aldrich, St. Louis, MO, USA) for 5 min at 37 °C. By adding culture media, the reaction was stopped, and the cell suspension was transferred into a 15 mL Falcon tube (Corning Incorporated; Tewksbury, MA, USA). After centrifugation for 5 min at 133× *g*, the cell pellet was resuspended in culture media and 200 µL were transferred into an Eppendorf cup (Eppendorf, Hamburg, Germany) for counting.

Cell counting was done with CASY Cell counter (OMNI Life Science, Bremen, Germany). 1 × 10^5^ were seeded in 6-well plates for stress protocols and 2–3 × 10^6^ were cultivated in T75 Flasks for further use. For freezing, the cell pellet was taken up in 1 mL of fibroblast freezing medium (PrimFibM, supplemented with 10% dimethyl sulfoxide (DMSO)) and transferred into 2 mL cryo store vials (Lab Solute by Th. Geyer; Renningen, Germany). An evenly freezing of the sample was guaranteed by transferring the vials into an isopropanol container and freezing it for short-term purposes at −80 °C. Long-term storage and biobanking was carried out at T < −180 °C using a SmartFreezer preservation system (Angelantoni Life Sciences, Milan, Italy).

For thawing, the fibroblasts in cryo vials were put shortly into the waterbath (37 °C) and transferred into a 15 mL Falcon tube with 5–10 mL of PrimFibM. The suspension was centrifuged for 5 min at 133× *g* in order to eliminate the cell-toxic DMSO. The cell pellet was resuspended in 10 mL of PrimFibM and seeded into a T75 flask.

### 2.3. Stress Protocols

The experiments were conducted under three different conditions: non-treated (N), treated with dexamethasone (DEX, 1 µM, 7 days), or with glucose-free/galactose (GAL)-containing medium (10 mM galactose, 7 days), to challenge the cells with either hormonal or metabolic stress, respectively.

### 2.4. Oxygen Consumption Rate (Respiration)

We seeded 3 × 10^5^ fibroblasts into the wells of the Agilent miniplates (Agilent Technologies; Santa Clara, CA, USA). One respiratory measurement included three technical replicates for each patient and control cell line of the same treatment (N, DEX or GAL). Wells B to D contained patient cells and wells E to G contained control cells, respectively vice versa. The wells A and H remained without cells as a blank control. A XFp Flux cartridge (Agilent Technologies; Santa Clara, CA, USA) was equilibrated with 200 µL in the wells A to H and 400 µL of Agilent Seahorse XF Calibrant solution (Agilent Technologies; Santa Clara, CA, USA) in the outer chambers and left at 37 °C in a non-CO_2_ incubator overnight. The following day, the miniplate wells containing the cells were washed with 200 µL of Seahorse Assay Medium (Seahorse XF Base Medium, Agilent Technologies; Santa Clara, CA, USA, supplemented with 10 mM glucose, 1 mM sodium pyruvate and 2 mM L-Glutamine (all Gibco by Life Technologies; Carlsbad, CA, USA), filled with 180 µL of Seahorse Assay Medium and left for 30 min to 1 h in a non-CO_2_ incubator.

Preparing the XFp Mito Stress Test kit: The component stocks were diluted 1:1000 in Assay Media and applied into the cartridge into different volumes in order to get a final concentration of 1 µM oligomycin (Cayman Chemical Company; Ann Arbor, MI, USA), 2 µM carbonyl cyanide-4-(trifluoromethoxy) phenylhydrazone (FCCP) (Cayman Chemical Company; Ann Arbor, MI, USA) and 0.5 µM rotenone/antimycin A (Cayman Chemical Company; Ann Arbor, MI, USA). The injection ports of the Seahorse XFp Flux cartridge were loaded with defined volumes of the respective compounds. 20 µL Oligomycin were applied into all of the A ports, 22 µL FCCP were pipetted into ports B and 25 µL of the rotenone/antimycin A mixture were put into the ports C in all of the wells A to H. After the experiment, the assay medium was aspirated, and the fibroblasts were fixed with 4% paraformaldehyde (PFA) (Carl Roth, Karlsruhe, Germany) for 10 min for normalization with Hoechst staining.

### 2.5. Normalization of Oxygen Consumption Rate (OCR) Values

The fixed cells were washed with 200 µL PBS and stained with Hoechst (Gibco by Life Technologies; Carlsbad, CA, USA, 1:1000 in PBS) for 10 min in the dark and washed three times with 200 µL PBS. Fluorescent nuclei were documented using a 5× objective lens (ECPlan-Neofluar, Zeiss, Jena, Germany) at a Zeiss Observer Z.1 microscope equipped with a SPOT RT3 camera (Diagnostic Instruments Inc, Sterling Heights, MI, USA). Images were further processed by adjusting the threshold, binary, and watershed settings/functions of the ImageJ software, and the number of nuclei/well were counted automatically. The cell numbers/well were entered into the Wave files, which were created by the XFp Flux Analyzer.

### 2.6. Luminescent Assay for ATP Content

1 × 10^5^ cells were pelletized in a 1.5 mL Eppendorf cup and stored at −20 °C if necessary. According to the manufacture’s advice, the CellTiter-Glo^®^Reagent containing CellTiter^®^Substrate and CellTiter^®^Buffer, was thawed on ice. As an ATP standard curve, concentrations of 10 µM, 100 nM, 10 nM, 10 pM ATP in PBS were used. The cell pellets were resuspended in 500 µL PBS and heated at 100 °C for 2 min in order to remove ATPases. Subsequently, the samples were stored on ice. 50 µL of each sample and each standard were applied to a black 96-well-plate in duplicates. We added 50 µL of the CellTiter-Glo^®^Reagent to the ATP standards and the samples and the 96-well plate was shaken for 2 min in the dark and the absorption was measured at the VarioScan with an integration time of 1 s. The relative light unit (RLU) generated by the SkanIT Software can be calculated to the actual ATP concentrations with the help of the ATP standard curve.

### 2.7. Mitochondrial Membrane Potential (JC-1)

We seeded 1.5 × 10^5^ fibroblasts on sterile glass coverslips (diameter 25 mm), placed in 6-well plates, and grown overnight in DMEM/Nutrient Mixture F-12 Ham, supplemented with 10% fetal calf serum and 1% antibiotic/antimycotic solution (Sigma) at 37 °C, humidified air and 5% CO_2_. The next day, cells were loaded with 200 nM JC-1/Pluronic (Life Technologies) in Opti-MEM (Life Technologies, Carlsbad, CA, USA) for 30 min at 37 °C, humidified air and 5% CO_2_. For imaging, coverslips were washed with assay buffer (140 mM NaCl, 5 mM KCl, 1.8 mM CaCl_2_, 1 mM MgSO_4_, 10 mM glucose, and 10 mM 4-(2-hydroxyethyl)-1-piperazineethanesulfonic acid (HEPES) and mounted in a chamber on the inverted microscope (ZEISS Observer Z.1, Jena, Germany). A Lambda DG4 high-speed wavelength switcher (Sutter instruments, Novato, USA) allowed the excitation of JC-1 at 480/36 nm. Cells were imaged using a Fluar 40/1.3 objective lens (ZEISS). The emitted light was filtered at 537/42 nm and 620/60 nm for green or red fluorescence, respectively, and finally detected by a charge-coupled device (CCD) camera (AxioCam MRm, ZEISS, Jena, Germany). Mitochondrial membrane potential was analyzed as ratio of red versus green fluorescence intensities in regions of interest, drawn over selected cells in the visual field using the Zen imaging software (ZEISS) and FIJI/ImageJ [12].

### 2.8. Imaging of Cytosolic Ca^2+^ (Fura-2/AM)

We seeded 1.5 × 10^5^ fibroblasts on sterile glass coverslips (diameter 25 mm), placed in 6-well plates, and grown overnight in DMEM/Nutrient Mixture F-12 Ham, supplemented with 10% fetal calf serum and 1% Antibiotic/Antimycotic Solution (Sigma) at 37 °C, humidified air and 5% CO_2_. The next day, cells were loaded with 2 µM Fura-2/AM and Pluronic F127 in OptiMEM (Life Technologies) for 30 min at 37 °C, humidified air and 5% CO_2_. For imaging, the loading medium was replaced by assay buffer (140 mM NaCl, 5 mM KCl, 1.8 mM CaCl_2_, 1 mM MgSO_4_, 10 mM glucose, and 10 mM HEPES) and the coverslip was mounted in a chamber on the inverted microscope (ZEISS Observer Z.1, Jena, Germany) equipped with a Fluar 40/1.3 objective lens (ZEISS). Cells were illuminated with light of 340 and 380 nm (BP 340/30 HE, BP 387/15 HE) using a fast wavelength switching and excitation device (Lambda DG-4, Sutter Instrument), and fluorescence was detected at 510 nm (BP 510/90 HE and FT 409) using an AxioCam MRm LCD camera (ZEISS). The cytosolic Ca^2+^ level was analyzed as fluorescence ratio at 510 nm after excitation at 340 and 380 nm. Regions of interest were drawn over selected cells in the visual field using the Zen imaging software (ZEISS) and FIJI/ImageJ.

### 2.9. gDNA Extraction

For each cell line, gDNA was extracted from two biological replicates. Genomic DNA (gDNA) was extracted from 1 × 106 cells using QIAmp DNA MiniKit (QIAGEN, Hilden Germany) following the manufacturer’s protocol. The DNA concentration was determined at a NanoDrop spectrophotometer and all samples were adjusted to 50 ng/µL for quantitative real-time polymerase chain reaction (RT-PCR) measurements.

### 2.10. Mitochondrial DNA (mtDNA) Copy Number

The mitochondrial DNA (mtDNA) copy number was evaluated by analyzing the ratio of the amount of mitochondrially encoded tRNA leucine 1 (mt-TL1) DNA to a reference nuclear single copy gene (beta-2 microtubulin, B2M). The primers for mt-TL-1 were as follow: F 5’-CACCCAAGAACAGGGTTTGT and R 5’-TGGCCATGGGTATGTTGTTA. The primers for B2M were: F 5’-TGCTGTCTCCATGTTTGATGTATCT and R 5’-TCTCTGCTCCCCACCTCTAAGT (Metabion, Planegg, Germany). Primer efficiency was tested using five times a threefold serial dilution of a DNA sample to obtain a standard curve. PCR efficiency E was obtained from slope of the resulting standard curves according to $E=10^{-1/slope}-1$. To improve precision, the samples were pipetted in quadruplicates. The qPCR is run in an initial phase of 5 min at 95 °C, followed by 45 cycles of 95 °C for 15 s and 60 °C for 30 s. The melting curve is assessed from 65 °C to 95 °C. Relative quantification was applied to calculate the number of mtDNA per diploid (2n) cell according to $2\times E^{-∆∆Cq}$, with Cq being the quantification cycle, ΔΔCq is Cq_mt_-Cq_nuc_, E is the averaged mean efficiency of the PCR reactions of the two targets. The mtDNA copy number was assessed from two biological replicates for each cell line and each biological replicate was measured in two separate runs [13].

### 2.11. Data Analysis and Statistics

Data collection and calculations were done with Microsoft EXCEL. Graphical depiction and statistical analysis were conducted with Graph Pad Prism 8.0.2 (GraphPad Software; San Diego, CA, USA). For all analysis, the mean of two to three technical replicates was calculated and two or three biological replicates were averaged. All measurements were conducted pairwise, e.g., a direct comparison of MDD vs. control was conducted. Data are presented as mean ± standard error of the mean (SEM), unless otherwise stated. All data are checked for normality distribution and consequently, the appropriate statistical tests are applied (paired t-test or Wilcoxon matched-pairs singed rank test). For multiple comparisons (oxygen consumption rate (OCR) vs. extracellular acidification rate (ECAR) for MDD vs. control) analysis of variance (ANOVA) with repeated measures was applied. *p*-value limit for statistical significance is set to ≤0.05. Correction for multiple comparison was applied if appropriate.

## 3. Results

### 3.1. Clinical and Descriptive Data of Patients and Control Subjects

Sixteen patients diagnosed with major depressive disorder and 16 healthy non-depressive controls participated in this study. Patients were asked to participate at the end of their inpatient stay at the Department of Psychiatry and Psychotherapy of the University of Regensburg. At this time, they received antidepressant medication (see Supplement Table S1) and showed improvement in the severity of depressive symptoms. The non-depressive control subjects were selected individually to match the gender and age of the corresponding patient.

To characterize the cohort of patients and control subjects, we surveyed the following parameters: Age, gender, body mass index (BMI), and Hamilton-rating scale for Depression (HAM-D21) (Table 1). As the controls were selected to match the gender and age of the individual patients, the groups do not differ in gender proportion (11 males and 5 females) and mean age (MDD 31 ± 3.12 years vs. Ctrl 32 ± 2.81 years; *p* = 0.682, paired *t*-test). Moreover, the groups were not different with regard to the BMI (MDD 23.0 ± 0.48 vs. Ctrl 24.2 ± 3.28; *p* = 0.161, paired *t*-test). Patients with major depressive disorder according to the ICD10, were rated with the HAM-D, which ranged from 20 to 34 (25.2 ± 4.4, mean ± SD), indicating a medium severe to severe depression. The non-depressive control group showed a HAM-D score ranging from 0–3, indicating the absence of depression at the time of the interview and skin biopsy extraction. Moreover, the controls did not report a history of depressive episode or other psychiatric disease. All participants gave written informed consent and all study procedures were approved by the ethics committee of the University of Regensburg (ref: 13-101-0271).

### 3.2. Mitochondrial Oxidative Phosphorylation System (OXPHOS)

In search for a phenotype in cells derived from depressed patients, we set out to investigate the bioenergetic core function of mitochondria, namely the ability to generate ATP by means of the mitochondrial oxidative phosphorylation system (OXPHOS). During this process, ATP is generated in a biochemical reaction primarily driven by the proton gradient (proton motive force, PMF), which is a result of sequential redox reactions at the inner mitochondrial membrane, in conjunction with the translocation of proteins into the intermembrane space (IMS). Finally, the consumption of molecular oxygen by accepting the electrons delivered by the ETC (reduction of oxygen to form water), can be used as a readout for the function and performance of the OXPHOS.

**Figure 1:**
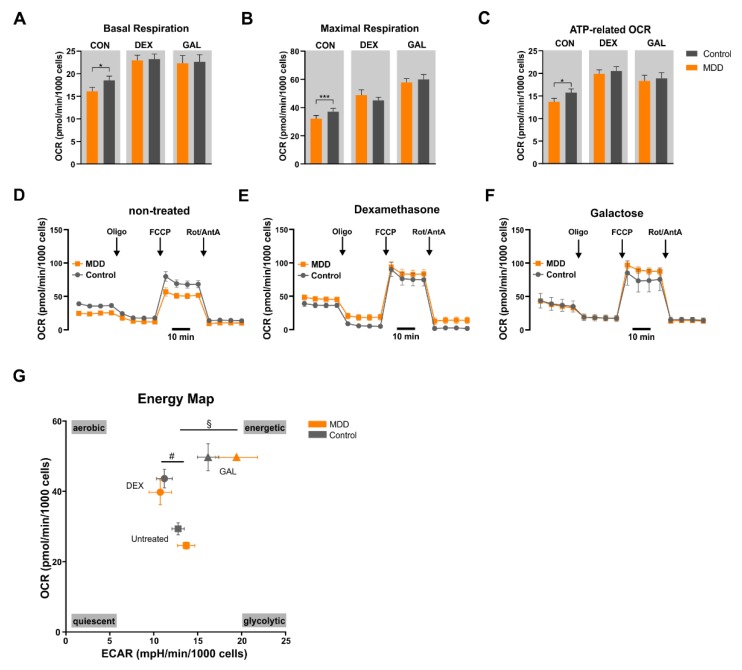
Oxygen consumption rates (OCR) (**A**–**G**) and extracellular acidification rate (ECAR) (**G**) measured by Seahorse XFp Flux Analyzer in major depressive disorder (MDD, indicated in orange) and control (indicated in grey) fibroblasts under non-treated conditions, as well as after one week of dexamethasone (DEX, 1 µM) or galactose (GAL) stress (10 mM GAL, glucose-free). The Mito Stress Test allows the analysis of basal (**A**) and maximal respiration (**B**), as well as oxygen consumption related to ATP production (**C**). Bar graphs show normalized mean OCR values + standard error of the mean (SEM); MDD *n* = 16, control *n* = 16. Significant differences between MDD and non-depressive controls are indicated with *. Mito Stress Test in fibroblasts (**D**–**F**). Exemplary curves for OCR measurement during the Mito Stress Test for MDD and control fibroblast lines (pair #6). Sequential injection of oligomycin, carbonyl cyanide-4-(trifluoromethoxy)phenylhydrazone (FCCP) and rotenone/antimycin A affects OCR by interaction with the electron transport chain (ETC) complexes ETC in the oxidative phosphorylation system (OXPHOS) of untreated (**D**), DEX-treated (1 µM, 7 days) (**E**) and GAL-stressed fibroblasts (glucose-free, 10 mM galactose, 7 days) (**F**). (**G**) Energy map of fibroblasts. Mean OCR in dependence of mean ECAR are shown for MDD and control fibroblasts for non-treated, DEX-treated (1 µM, 7 days) and GAL-stressed (glucose-free, 10 mM galactose, 7 days) conditions. Significant effects of treatment were found for DEX (# *p* ≪ 0.05, compared with non-treated, analysis of variance (ANOVA) with repeated measures, Greenhouse–Geisser correction, post-hoc analysis with Bonferroni) and GAL (§ *p* ≪ 0.05, compared with non-treated, ANOVA with repeated measures, Greenhouse–Geisser correction, post-hoc analysis with Bonferroni), Data are shown as mean OCR ± SEM vs. mean ECAR ± SEM; MDD *n* = 16, control *n* = 16.

We used the Seahorse Flux Analyzer technology to directly measure the oxygen consumption in the presence of specific substrates and selective enzyme inhibitors, which can be instrumentalized to reveal the activity and capacity of the individual molecular respiratory complexes (Figure 1 and Supplemental Figure S1). To this end, the function of the ETC and OXPHOS in mitochondria of both MDD and control fibroblasts were assessed and compared. In detail, we analyzed the basal respiration, the oxygen consumption related to ATP synthesis, and the maximal respiration in the presence of the uncoupler FCCP of fibroblasts derived from depressed patients or non-depressed controls. We found a significantly lower basal respiration in MDD fibroblasts (16.09 ± 0.88 pmol/min/1000 cells) compared with fibroblasts derived from healthy control subjects (18.53 ± 0.95 pmol/min/1000 cells; *p = 0.02, paired *t*-test) (Figure 1A). Moreover, the oxygen consumption during maximal respiration also was significantly lower in MDD fibroblasts (32.20 ± 2.33 vs. 37.01 ± 2.4 pmol/min/1000 cells; *p = 0.0003, paired *t*-test) (Figure 1B). Additionally, the oxygen consumption related to ATP synthesis differs significantly in MDD versus control fibroblasts. MDD fibroblasts showed 13.71 ± 0.76 pmol/min/1000 cells, whereas healthy control fibroblasts presented 15.72 ± 0.82 pmol/min/1000 cells (*p = 0.026, paired *t*-test) (Figure 1C). These findings are in line with the hypothesis that mitochondria in cells from depressed patients possess a lower bioenergetic activity and demonstrate that this functional difference is evident in peripheral non-neuronal cells.

In order to investigate whether fibroblast mitochondria from MDD patients or healthy controls respond differently to stress, cells were grown for seven days in glucose-free/galactose-containing medium or treated with 1 µM dexamethasone (7 days) to induce metabolic or hormonal stress, respectively. Each of the two stressors led to an increased basal respiration in both control and MDD fibroblasts, although the oxygen consumption rates were not different in the two cohorts under stress (Figure 1A). The maximal (uncoupled) respiration of control fibroblasts (37.01 ± 2.40 pmol/min/1000 cells) was substantially increased by treatment with dexamethasone (45.02 ± 2.32 pmol/min/1000 cells) and to an even higher extent when cells were grown under glucose-free/galactose-containing medium (60.04 ± 3.51 pmol/min/1000 cells). However, the oxygen consumption rates of MDD fibroblasts under stress were not significantly different from values of stressed control cells (Figure 1). Our findings indicate that the significantly lower bioenergetic activity of MDD fibroblasts is not evident any more under conditions of hormonal or metabolic stress. Nevertheless, the treatments with DEX and GAL induced significant changes in the metabolic parameters OCR and ECAR in MDD fibroblasts and control fibroblasts compared to the non-treated condition (Figure 1G).

### 3.3. ATP Content

**Figure 2:**
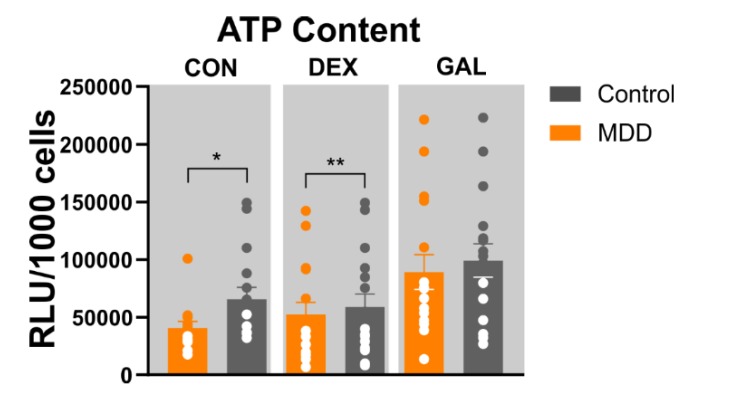
ATP content in fibroblasts. ATP content in MDD and control fibroblasts under non-treated, DEX-treated (1 µM, 7 days) or GAL-stressed conditions. Significant differences were found for MDD vs. control, non-treated (* *p* < 0.05, Wilcoxon matched-pairs signed rank test) and DEX (** *p* < 0.01, compared with control, Wilcoxon matched-pairs signed rank test). Bar graphs show normalized mean RLU values ± SEM. Dots show the distribution of single RLU values for MDD and control fibroblast lines; MDD *n* = 16, control *n* = 16.

Mechanisms affecting the oxidative phosphorylation in the respiratory chain should also directly affect the provision of energy. Thus, we investigated the energetic capacity of fibroblasts derived from MDD patients and healthy controls by direct measurement of the ATP concentration in the different cell populations. The ATP production in MDD and control fibroblasts were assessed by a bioluminescence-based assay under control conditions as well as under metabolic (glucose-free/galactose) or hormonal (dexamethasone) stress. Following our hypothesis that cells derived from depressed patients present a reduced bioenergetic status, we found a significantly reduced ATP level in MDD fibroblasts compared to healthy controls) (*p = 0.036, *n* = 16, Wilcoxon matched-pairs signed rank test) (Figure 2). During stress conditions, the ATP level of dexamethasone-treated MDD fibroblasts (1 µM dexamethasone, 7 days) was significantly lower than in dexamethasone-treated control cells (*p = 0.0052, *n* = 16, Wilcoxon matched-pairs signed rank test), whereas metabolic stress (10 mM galactose, 7 days) abolished the significant differences between MDD fibroblasts and healthy controls (*p* = 0.463, *n* = 16, Wilcoxon matched-pairs signed rank test). Moreover, fibroblasts derived from depressed patients showed a significantly higher ATP content subsequent to GAL stress compared to the non-treated condition (*p = 0.0468, Mann–Whitney U test, *n* = 16) whereas DEX did not cause a significant alteration in the ATP content (Figure 2). These findings indicate that cells derived from depressed patients show a stronger response to the metabolic challenge than fibroblasts derived from healthy controls.

### 3.4. Mitochondrial Membrane Potential (JC-1)

As a chemo-electrical potential difference between the mitochondrial matrix and the intermembrane space (IMS), the mitochondrial membrane potential (MMP) is generated by consecutive redox reactions associated with the translocation of electrons and protons over the inner mitochondrial membrane (IMM) by the activity of the ETC. The resulting proton gradient is the primary driving force for the synthesis of ATP, and at the same time is diminished again by the backflow of protons into the matrix through the active ATP synthase. Thus, the MMP serves as an indicator for the bioenergetic state of mitochondria and may indicate bioenergetic disbalance resulting from mitochondrial dysfunction [14].

**Figure 3:**
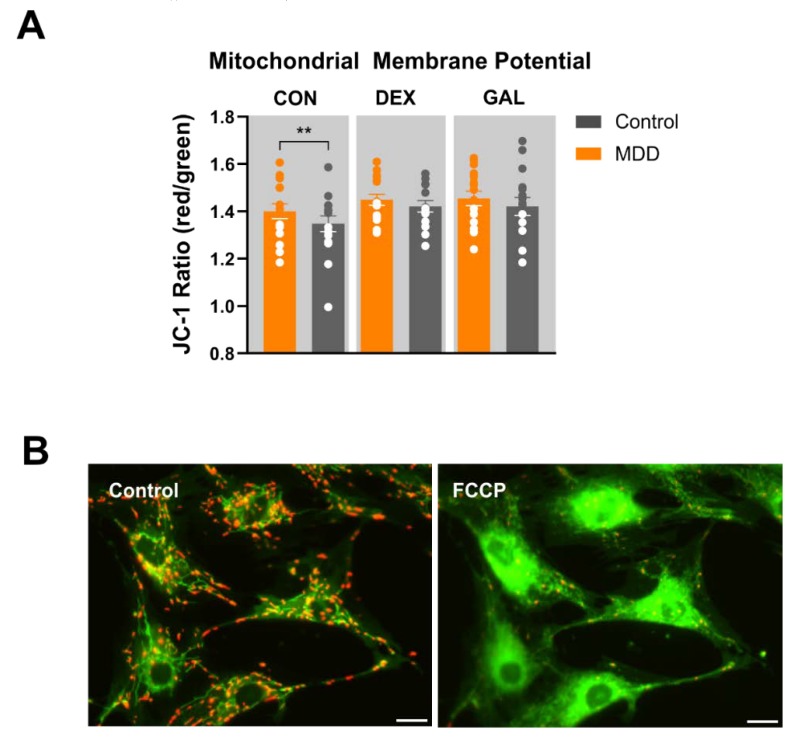
Mitochondrial membrane potential of fibroblasts. (**A**) Red/green (JC-1 aggregate/monomer) ratios of MDD and control fibroblasts under non-treated, DEX-treated (1 µM, 7 days), or GAL-stressed conditions (glucose-free, 10 mM galactose, 7 days). Significant differences were found for MDD vs. control, non-treated (* *p* < 0.05, compared with control, Student’s *t*-test, paired, two-tailed). Bar graphs show mean red/green ratios ± SEM, MDD *n* = 16, control *n* = 16). Dots show the distribution of single red/green values for MDD and control fibroblast lines under the indicated conditions; MDD *n* = 16, control *n* = 16. (**B**) Fluorescence microscopy image of fibroblasts loaded with the cationic dye JC-1 under basal conditions (left) and after treatment with 20 µM FCCP to uncouple the proton gradient leading to depolarization of the MMP. Aggregates of the dye fluoresce red, monomers fluoresce green. Scale bar indicates 20 µM.

The MMP of cultured human skin fibroblasts was analyzed by loading the cells with the fluorescent dye JC-1. The cationic dye accumulates in the mitochondrial membranes to an extent, which is dependent on the strength of the electric field. In negatively charged (highly energized) mitochondria, JC-1 molecules form red fluorescing aggregates, while the fluorescence changes to green, when the dye molecules disaggregate into monomers in response to dissipation of the transmembrane potential. The ratio of the fluorescence signals emitted by the two states of JC-1 is then analyzed as a measure of the MMP. Under untreated, non-stressed conditions, the aggregate/monomer fluorescence ratio differed significantly in the fibroblasts of the two groups (Figure 3). Compared to control fibroblasts, MMD cells showed a higher fluorescence ratio (MMD 1.40 ± 0.0008 vs. Cntrl 1.35 ± 0.0008; *p < 0.01, *t*-test, paired), indicating that fibroblasts of depressed patients showed a more negative (hyperpolarized) MMP. Considering our results showing a lower oxygen consumption rate (Figure 1) and lower ATP content in MMD fibroblasts (Figure 3), this observation is in line with a lower proton leak in MDD fibroblasts (Supplemental Figure S1). The more negative MMP is suggested to be a consequence of a reduced number of protons returning to the matrix independently from the ATP synthase. However, a difference in MMP was not observed under stressed conditions. Treatment of skin fibroblasts with dexamethasone (1 µM, 7 days) or galactose (10 mM, glucose-free for 7 days) led to only minor differences in MMP between the groups (DEX: MMD 1.45 ± 0.006 vs. Cntrl 1.42 ± 0.006; *p* = 0.187, *t*-test, paired; Gal: MDD 1.46 ± 0.008 vs. Cntrl 1.42 ± 0.009; *p* = 0.244, *t*-test, paired) (Figure 3).

### 3.5. Ca^2+^ Homeostasis

**Figure 4:**
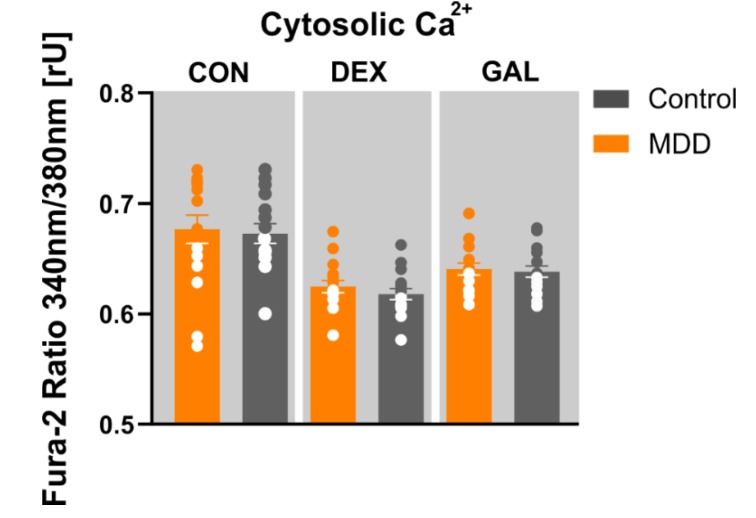
Cytosolic Ca^2+^ homeostasis in fibroblasts. Shown are the Fura-2 340 nm/380 nm ratios of MDD and non-depressed control fibroblast lines under non-treated, DEX-treated (1 µM, 7 days) or GAL-stressed conditions (glucose-free, 10 mM galactose, 7 days). No significant differences were found. Bar graphs show mean ratios (340 nm/380 nm; ratios ± SEM). Dots show the distribution of ratios (340 nm/380 nm) for MDD and control fibroblast lines under indicated conditions (MDD *n* = 16, control *n* = 16).

Since the MMP is one of the functional variables/parameters involved in regulating cellular Ca^2+^ homeostasis, we set out to investigate cytosolic Ca^2+^ levels in the different groups by loading the cells with the Ca^2+^ sensitive dye Fura-2/AM. Although we found a significant difference in the MMP between the groups in non-stressed cells, we did not detect between-group differences in cytosolic Ca^2+^ levels (Figure 4). The fluorescence ratio (F_340 nm_/F_380 nm_) was 0.67 ± 0.013 in non-stressed MDD fibroblasts and 0.67 ± 0.009 in corresponding controls (*p* = 0.495, Wilcoxon matched-pairs signed rank test). Hormonal (dexamethasone, 1 µM, 7 days) or metabolic stress (10 mM galactose, glucose-free, 7 days) did not affect cytosolic Ca^2+^ levels in MDD fibroblasts, nor in control cells (DEX: MDD 0.64 ± 0.005 vs. Cntrl 0.64 ± 0.006; *p* = 0.229 t-test, paired; Gal: MDD 0.62 ± 0.005 vs. Cntrl 0.62 ± 0.006; *p* = 0.665, *t*-test, paired).

### 3.6. Mitochondrial Mass/mtDNA Copy Number

**Figure 5:**
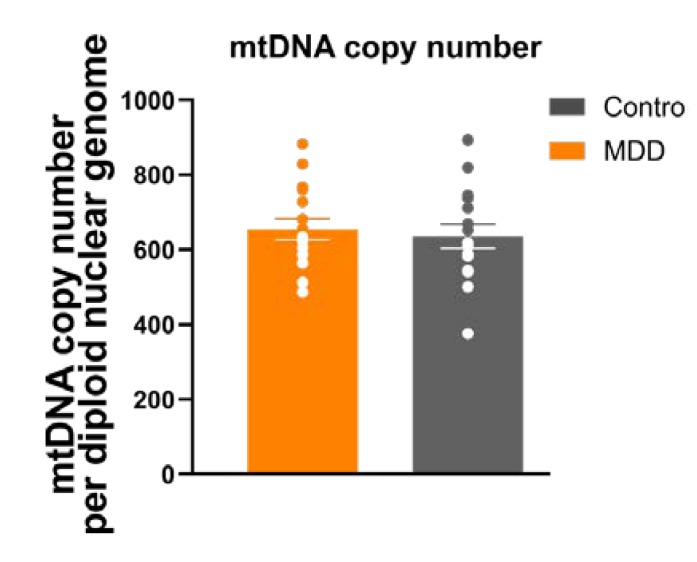
Mitochondrial DNA (mtDNA) copy number per nDNA in 16 MDD patient and control fibroblast cell lines. No significant differences were found. Bar graph show mean mtDNA copy number ± SEM. Dots show the single values of mtDNA copy numbers for MDD and control fibroblast lines; MDD *n* = 16, control *n* = 16.

The mitochondrial content of a cell is an indicator of the cellular energy demand and is potentially disturbed by an imbalanced metabolism [15]. In order to evaluate whether our observed difference in ATP content and respiratory activity (oxygen consumption rate, OCR) in MDD fibroblasts is associated with an altered function of the ETC and/or ATP synthase, or whether this phenotype is related to a lower mitochondrial content, we set out to analyze the content of mitochondrial DNA (mtDNA copy number) as a quantitative measure of mitochondrial mass in MDD and control fibroblasts. By means of qPCR, we quantified mtDNA copy number by targeting the mitochondrial t-RNA^Leu^ gene (mitochondrially encoded tRNA leucine 1) and related it to the single copy nuclear gene beta-2-microglobulin [13]. We found no significant differences in mtDNA copy number between fibroblasts of depressed patients compared to non-depressed controls (MDD 654 ± 28.04 vs. 636 ± 31.68; *p* = 0.654, *t*-test, paired) (Figure 5). Based on these results, our data are in favor of a mitochondrial dysfunction in MDD fibroblasts rather than a difference in mitochondrial content.

## 4. Discussion

The bioenergetic status of a cell can be regarded as an indicator of mitochondrial function. In addition, it is dependent on the cellular mitochondrial content. Mitochondrial dysfunction might be related to defects within the electron transport complexes and/or the ATP-synthase, as well as to the assembly and dynamic of mitochondrial supercomplexes [14]. The disturbance of the mitochondrial energy supply, either as a cause or consequence of depression, may contribute to the molecular pathophysiology of depressive symptoms, possibly by inducing sickness behavior and increased inflammatory states [16]. In the present study, we could show that mitochondria-related bioenergetic functions in fibroblasts derived from depressed patients (MDD) are different in several aspects from gender- and age-matched control cells.

### 4.1. Mitochondrial Respiration

Fibroblasts from MDD patients show lower oxygen consumption rates (OCR) for basal and maximal respiration, spare respiratory capacity, non-mitochondrial respiration and ATP-turnover related respiration. Lower basal respiration and a reduced ATP-related OCR could in general be a consequence of a reduced activity or malfunction of the mitochondrial respiratory chain, or due to a restricted substrate or enzyme availability. A restricted availability of glucose or fatty acids, as well as a dysfunction of metabolic enzymes such as hexokinase or acyl-CoA dehydrogenase, reduces the availability of the reduction equivalents NADH/H^+^ and FADH_2_, which are needed to fuel the ETC [17]. However, a lower basal respiration may also result from a reduced function of the ETC itself, reflecting a lower activity of the complexes I-IV in MDD fibroblasts. In addition, a reduced respiratory activity might also be a consequence of a lower mitochondrial content in MDD fibroblasts (see below).

Non-mitochondrial oxygen consumption is observed at low levels in a variety of cells and tissues. It has been linked to an inefficient ETC or to other cellular oxidative reactions, which are unrelated to energy metabolism [18]. We observed that MDD fibroblasts exhibit a lower non-mitochondrial respiration compared to control fibroblasts, which either points to a reduced activity of the ETC or a generally decreased metabolism.

The reduced maximal (uncoupled) respiration along with the lower spare respiratory capacity demonstrates that fibroblasts of MDD patients exhibit a reduced capacity of mitochondrial respiration. This decreased capacity might result in a lack of energy in times of higher energy demand [19].

Similar observations to those in the present study were made in peripheral blood mononuclear cells (PBMCs) of acutely depressed patients. Karabatsiakis et al. also observed a reduction in routine respiration, ATP turnover-related respiration and coupling efficiency [9]. These findings also are in line with Hroudová and coworkers [10]. They demonstrated that the physiological respiratory rate and the maximal capacity of the ETC are significantly decreased in intact blood platelets of patients with a current depressive episode. Another study figured out that muscle cells of depressed patients show an impaired activity of complexes I+III and II+III. They also discovered a significant decrease of mitochondrial ATP production rates [8]. This is in agreement with our study showing significantly lower ATP levels in MDD fibroblasts compared to controls. In general, our findings are in line with an overall decreased respiration of MDD fibroblasts and a reduced ATP content.

### 4.2. Mitochondrial Content

Mitochondrial content is an important quantitative measure, since the mitochondrial mass is dependent on intact mitochondrial dynamics and influences mitochondrial bioenergetics [20,21]. In the present study, we analyzed the mtDNA copy number as a quantitative measure for mitochondrial mass or density [13,22,23] and could not detect significant differences between MDD fibroblasts and non-depressive controls. This finding suggests that the altered respiratory parameters and bioenergetic properties, which we found in MDD fibroblasts cannot be attributed to differences in the mitochondrial mass. In line with this, Hroudová and coworkers reported significantly reduced respiration and capacity of electron transport in blood platelets of depressed patients. The functional parameters were normalized to the platelets’ mitochondrial content by their citrate synthase activity [10]. However, PBMCs from depressed subjects showed a higher mitochondrial content, which could be interpreted as an attempt to compensate for the reduced efficiency in mitochondrial ATP production [9]. Such a compensatory mechanism to balance bioenergetic deficiencies was also proposed by Wang and coworkers [24]. Another study revealed that the amount of mtDNA alters in response to external stress. The level of mtDNA increased significantly in the saliva and blood of patients suffering from MDD compared to controls. Moreover, chronic stress also altered the amount of mtDNA in mouse tissues, paralleled by a lowered mitochondrial respiratory activity [25,26].

### 4.3. Mitochondrial Membrane Potential

We found that the MMP is more negative in MDD fibroblasts and that these cells possessed a lower ATP level compared to fibroblasts from non-depressive controls. This observation might be related to a reduced function of the ATP synthase, which causes an accumulation of protons leading to a steeper gradient and thus to a higher (i.e., more negative) MMP. The general assumption is that the electrochemical gradient regulates the activity of the ETC complexes. At high potentials further proton pumping is impaired. A decrease of the MMP due to proton utilization, e.g., by the ATP synthase, allows the ETC to rebuild the MMP. Mitochondria in intact cells respire between the extreme energetic states, state 3 in the presence of ADP and state 4 when ADP has been converted into ATP [27]. Studies of fibroblasts with primary defects in mitochondrial ATP synthase show that the MMP at state 4 is normal, but ADP-induced discharge of the MMP is impaired as ATP synthesis at state 3 is decreased. Increased MMP and low ATP synthesis is also found when the ATP synthase content is diminished by altered biogenesis of the enzyme complex [28].

### 4.4. Ca^2+^ Homeostasis

Mitochondria essentially contribute to the cellular Ca^2+^ homeostasis. They accumulate Ca^2+^ in an energy-dependent way, mainly driven by their negative membrane potential, and they release Ca^2+^ through antiporters. Mitochondrial key functions—in particular the matrix dehydrogenases—are strongly influenced by the cytosolic Ca^2+^ level [29]. Cytosolic Ca^2+^ also influences the activity of other mitochondrial enzymes at the IMM such as the glycerophosphate dehydrogenase or the malate-aspartate shuttle [30]. The analysis of cytosolic Ca^2+^ in MDD fibroblasts and fibroblasts from controls did not reveal any significant differences. The more negative MMP in MDD fibroblasts might cause a higher uptake of Ca^2+^ into the mitochondrial matrix. Exceeded Ca^2+^ uptake by mitochondria is able to trigger a bioenergetic failure of the organelle through the opening of the permeability transition pore (PTP), and the release of Cyt c along with other pro-apoptotic factors, which cause cellular death by apoptosis or necrosis [31]. Since mitochondrial Ca^2+^ levels were not assessed in the present study, this analysis will be an important objective in the future.

### 4.5. Impact of Hormonal or Metabolic Stress on Mitochondrial Function

In order to assess whether fibroblasts derived from depressed patients or non-depressed controls responded differentially to stress, we challenged the cells with dexamethasone or with galactose-containing and glucose-free medium for one week, thereby evoking hormonal or metabolic stress, respectively. Although glucocorticoids play a beneficial role in acute stress by regulating physiological processes related to fight or flight behavior [32,33], chronic glucocorticoid treatment induces serious side effects. Pathologies are related to insulin resistance and catabolic effects on skeletal muscle, and are associated with numerous physical diseases as well as with mental disorders [34,35]. It has been reported that chronic glucocorticoid treatment causes ETC dysfunction, increased levels of reactive oxygen species, mitochondrial abnormalities, apoptosis and cell death [36]. In our study, we could show that both dexamethasone and galactose treatment increased basal and maximal respiration, whereas the difference between depressive and non-depressive fibroblasts, which we observed under non-stress conditions vanished under stress (Figure 1).

It has been already shown that dexamethasone (1 µM, 48 h) caused impaired insulin-induced glucose uptake and mitochondrial dysfunction, which became manifest by decreased intracellular ATP and MMP, increased intracellular and mitochondrial levels of reactive oxygen species (ROS), as well as by elevated mtDNA damage [37]. The authors observed changes in mitochondrial dynamics and biogenesis and related their findings to decreased *Drp1*, increased *Mfn2*, and decreased *PGC-1*α, *NRF1*, and *TFam* levels. In our studies, we found that treatment with DEX resulted also in lower ATP levels, but showed higher OCR in both MDD and control fibroblasts when compared to untreated cells. This finding supports a scenario in which the chronic treatment of fibroblasts with DEX (1 µM) seems to have rather adverse effects on the energy supply in spite of increased respiratory activity. The reductive effect of DEX on ATP levels were significantly stronger in MDD fibroblasts compared to controls. However, the stimulatory effect of DEX on the ETC might lead to increased MMP in both MDD and control fibroblasts, ablating the difference observed in untreated cells.

Metabolic stress on fibroblasts was induced by galactose (10 mM) replacing for glucose in the culture medium. Under this condition, the cells are forced to have an increased reliance on OXPHOS for their energy supply [38,39], since galactose will not deliver any net ATP during glycolytic metabolism [40]. Galactose-induced stress is known to modulate mitochondrial structure and increase oxidative capacity by increased expression and elevated activity of enzymatic complexes [41]. Skin fibroblasts derived from patients with mitochondrial deficiency (e.g., cytochrome oxidase deficiency, complex I deficiency, pyruvate dehydrogenase complex deficiency or with multiple respiratory chain defects) were not able to survive when cultured in a galactose-based medium [39]. However, in the present study using MDD and control fibroblasts, GAL treatment resulted in an overall significantly increased metabolism compared to the non-treated state. Both OCR and ECAR were increased due to GAL treatment. Although the acidification rate (ECAR) is widely used to describe glycolytic events, extracellular acidification is also dependent on CO_2_ produced in the citric acid cycle in the context of OXPHOS. CO_2_ in aqueous solution will form H_2_CO_3_ and dissociate into HCO_3_^-^ + H^+^ thereby contributing to respiratory acidification [42].

Overall, metabolic stress in the form of glucose-free GAL exposure did not decipher different respiratory properties between patients and controls, except the non-mitochondrial respiration, which might be unrelated to mitochondrial function [18]. We found ATP levels higher for both groups compared to non-treated conditions although levels were not different between MDD fibroblasts and controls. We also did not detect significant differences in MMP or cytosolic Ca^2+^ in response to GAL exposure. However, Garbett et al. have shown that metabolic challenges evoked by substitution of glucose with GAL or reducing the abundance of lipids in the growth media of fibroblasts from MDD patients resulted in changes of mRNA and miRNA expression compared to fibroblasts from non-depressive controls. These stress-induced changes have been suggested to be MDD-related [7].

In conclusion, our data suggest a mitochondrial dysfunction, which led to a bioenergetic deficit in MDD, and we propose that skin fibroblasts as peripheral cells are involved in and associated with the manifestation of a psychiatric disorder.

## Figures and Tables

**Table 1:** Personal and clinical parameters of study participants.

Variables	Groups	
	MDD (*n* = 16)	Controls (*n* = 16)
**Age** (mean ± standard deviation (SD), years)	31 ± 3.12	32 ± 2.81
**Sex** (male, *n* (%)/female, *n* (%))	11 (69%)/5 (31%)	11 (69%)/5 (31%)
**Body mass index** (**BMI**, mean ± SD, kg/m^2^)	23.0 ± 0.48	24.2 ± 3.38
**Hamilton-ratingscale for Depression** (**HAM-D**, mean ± SD)	25.2 ± 4.4	--
**HAM-D @Time of Biopsy** (mean ± SD)	10.8 ± 1.9	

## Supplementary Materials

The following are available online at https://www.mdpi.com/2073-4409/9/4/884/s1, Figure S1: Oxygen consumption rates (OCR) measured by Seahorse XFp Flux Analyzer in MDD (indicated in orange) and control (indicated in grey) fibroblasts under non-treated conditions, as well as after one week of DEX (1 µM) or GAL stress (10 mM GAL, glucose-free), Table S1: Additional information on medication and hospitalization of MDD patients.
