# Supplementary material for: Major Depressive Disorder is Associated with Impaired Mitochondrial Function in Skin Fibroblasts
Source: Cells. 2020 Apr 4;9(4):884. doi: 10.3390/cells9040884 (PMC7226727; doi:10.3390/cells9040884)
Supplement: Supplementary file 1 [file cells-09-00884-s001.zip › Supl Table Patient Data CW.docx]

| **Pseudonym Patient ID** | **First diagnose of MDD [year of life]** | **Treatment before hospitalization** | **Treatment during hospitalization** | **Duration of hospitalization [days]** |
| --- | --- | --- | --- | --- |
| MDD #1 | 48 | untreated | Mirtazapine | 38 |
| MDD #2 | 35 | Citalopram (75 weeks)  Opipramol | Venlafaxin | 37 |
| MDD #3 | 17 | untreated | Duloxetin, Olanzapine | 107 |
| MDD #4 | 35 | Duloxetine, Mirtazapine, Pregabalin, Sertraline, Reboxetine, Olanzapine;  untreated for 2 years before hospitalization | Venlafaxin, Olanzapine | 38 |
| MDD #5 | 14 | untreated | Bupropion, Mirtazapine | 62 |
| MDD #6 | 21 | untreated | Escitalopram | 43 |
| MDD #7 | 23 | untreated | Escitalopram, Mirtazapine | 48 |
| MDD #8 | 41 | -2013: Trimipramine, Agomelatine, Mirtazapine, Pregabalin  2013-2015: Amitryptyline, Sertralin  2015-2016: Doxepin | Doxepin | 39 |
| MDD #9 | 18 | untreated | Trimipramine, Bupropion | 56 |
| MDD #10 | 21 | untreated | Agomelatine | 59 |
| MDD #11 | 21 | untreated | Escitalopram | 68 |
| MDD #12 | 22 | untreated | Escitalopram, Promethazin | 86 |
| MDD #13 | 23 | untreated | Escitalopram | 47 |
| MDD #14 | 16 | 2015-2016: Citalopram, Mirtazapine | Agomelatine | 50 |
| MDD #15 | 27 | untreated | Agomelatine | 54 |
| MDD #16 | 32 | 2015: Citalopram (6 month) | Escitalopram | 37 |

Supplemental Table 1: Additional information on medication and hospitalization of MDD patients
