# Supplementary material for: Major Depressive Disorder is Associated with Impaired Mitochondrial Function in Skin Fibroblasts
Source: Cells. 2020 Apr 4;9(4):884. doi: 10.3390/cells9040884 (PMC7226727; doi:10.3390/cells9040884)
Supplement: Supplementary file 1 [file cells-09-00884-s001.zip › Supplemental Figure 1 Legend.docx]

**
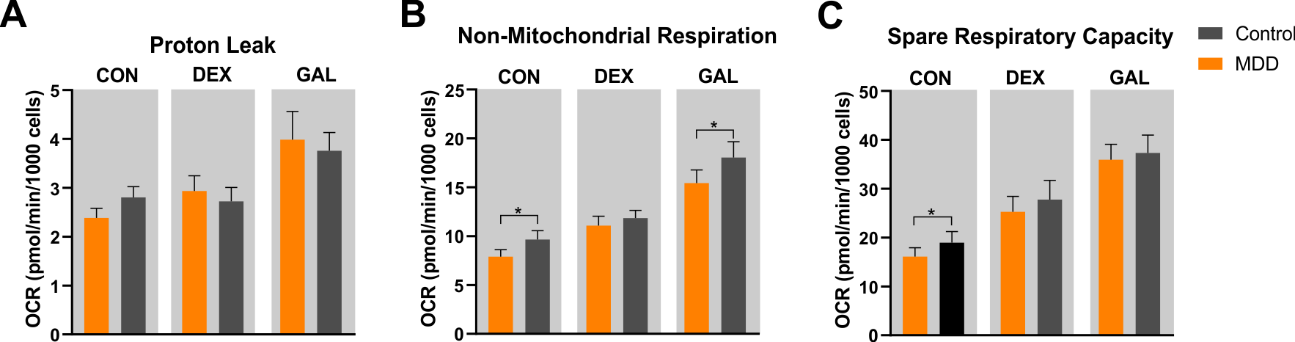
**

**Supplemental Figure 1** Oxygen consumption rates (OCR) measured by Seahorse XFp Flux Analyzer in MDD (indicated in orange) and control (indicated in grey) fibroblasts under non-treated conditions, as well as after one week of DEX (1 µM) or GAL stress (10 mM GAL, glucose-free). The Mito Stress Test allows, among others (see Figure 1), the analysis of the proton leak (**A**), and the non-mitochondrial respiration (**B**). The spare respiratory capacity (**C**) presents the metabolic reserve. Bar graphs show normalized mean OCR values + SEM; MDD n=16, control n=16. Significant differences between MDD and non-depressive controls are indicated with *.
